# Cross-Cultural Adaptation of the CHAMPS Questionnaire in French Canadians with COPD

**DOI:** 10.1155/2016/9304505

**Published:** 2016-05-31

**Authors:** Susanne Mak, Judith E. Soicher, Nancy E. Mayo, Sharon Wood-Dauphinee, Jean Bourbeau

**Affiliations:** ^1^School of Physical & Occupational Therapy, McGill University, Montreal, QC, Canada H3G 1Y5; ^2^Respiratory Epidemiology & Clinical Research Unit, McGill University, Montreal, QC, Canada H3H 2R9

## Abstract

Physical activity is difficult to measure in individuals with COPD. The Community Healthy Activities Model Program for Seniors (CHAMPS) questionnaire demonstrated strong clinometric properties when used with the elderly and with those affected by chronic disease. Study objectives were to translate, culturally adapt the CHAMPS into French, and reexamine its test-retest reliability and construct validity in French and English Canadians with COPD. This paper presents the cross-cultural adaptation of the CHAMPS; results of its clinometric testing will be described in another article. The CHAMPS examines the degree of physical activity performed in a typical week through two summary scales, caloric expenditure and activity frequency. The CHAMPS was only in English; thus, a cross-cultural adaptation was needed to translate the CHAMPS into French for use in French Canadians with COPD. Cross-cultural adaptation consisted of forward and back translation, with expert review at each stage of translation: minor inconsistencies were uncovered and rectified. Five French participants with COPD completed the finalized Canadian French CHAMPS and participated in cognitive debriefing; no problematic items were identified. A structured and stepwise, cross-cultural adaptation process produced the Canadian French CHAMPS, with items of equivalent meaning to the English version, for use in French Canadians with COPD.

## 1. Introduction

In patients with Chronic Obstructive Pulmonary Disease (COPD), amount of physical activity has been shown to be a strong predictor of mortality [1] and of hospital readmission [2]. Patients with COPD, however, often experience a decreased ability to engage in sustained physical activity [2–4] and thus have exhibited lower functional and respiratory capacities [5].

To understand the extent to which COPD affects physical activity level, it is necessary to measure physical activity by identifying and quantifying the types of activity in which COPD patients engage [6]. Methods include doubly labelled water and calorimetry that measure energy expenditure, as well as wearable monitors such as heart rate monitors, pedometers, accelerometers, and multiple sensor systems that provide estimates of different types and intensities of physical activity [7–10]. Although these methods provide easily quantifiable results, they may be expensive and cumbersome or are only usable in specific conditions [2, 8, 9, 11–13]. For some wearable monitors, the clinometric properties (e.g., reliability) have not been fully investigated [13].

An alternative method of measuring physical activity is the use of physical activity questionnaires with strong clinometric properties [14]. Questionnaires are less likely than wearable monitors to modify an individual's behaviour, are more convenient, and cost less [2, 7]. Depending upon the questionnaire items, it may not be possible to capture all relevant types of physical activity or their amount. For two questionnaires used extensively in physical activity research, the Stanford Seven-Day Physical Activity Recall Questionnaire [15] and the Yale Physical Activity Survey [16], study results demonstrated the questionnaires' ability to discriminate COPD patients by activity level, but the questionnaires were unable to determine the specific amounts of physical activity performed at an individual level.

The CHAMPS questionnaire is a self-administered questionnaire where the respondent must recall the type and frequency of physical activities he/she has engaged in on a weekly basis during the past month. An example of a questionnaire item is the following: “In a typical week during the past 4 weeks, did you walk leisurely for exercise or pleasure?” The frequency of the activity is estimated when the participant indicates how many times this activity was performed in a week. For the amount of physical activity, the participant considers the total hours per week spent on this activity and chooses one of six response options, ranging from less than one hour to 9 or more hours. Two summary measures of physical activity, caloric expenditure and frequency, are generated for all activities (any MET value) and activities of at least moderate intensity (MET value ≥ 3.0) [17].

In the original CHAMPS study, it was shown that the CHAMPS was able to distinguish individuals of varying physical activity levels, in which 10% of the study sample reported having a chronic respiratory condition [17]. Further clinometric testing of the CHAMPS has demonstrated promising results in COPD patients: moderate-to-high levels of test-retest reliability (ICCs = 0.58–0.67) [17] and low-to-moderate values of construct validity for the Physical Functioning scale of the SF-36 (*r* = 0.39 to 0.41) [18]. Preliminary work at our centre has provided further evidence of the CHAMPS' clinometric properties in COPD patients: the caloric expenditure and frequency measures of the CHAMPS differentiated COPD patients from healthy individuals [19]. Correlations between caloric expenditure and variables of importance in COPD, such as pulmonary function, health-related quality of life, and maximal exercise capacity, were demonstrated in a pilot study (*r*_*s*_ = 0.5–0.75) [19]. The frequency measure of the CHAMPS also correlated with health-related quality of life and maximal exercise capacity (*r*_*s*_ = 0.52–0.56) [19]. In addition, the CHAMPS questionnaire includes standardized instructions, is easily understood by COPD patients, and takes less than 20 minutes to complete. The CHAMPS questionnaire therefore offers promising applicability in both clinical and research settings.

For use in a French Canadian COPD population, translation of the questionnaire into Canadian French was needed [20]. A cross-cultural adaptation process was undertaken, in which the CHAMPS items were translated linguistically and also examined culturally to maintain content validity for French Canadians [20]. A Canadian French version of the CHAMPS will allow the assessment of physical activity patterns specific to this population, which will eventually contribute to the development of interventions promoting physical activity, ultimately having beneficial effects on function and morbidity. The objective of this study was to translate and culturally adapt the CHAMPS for use in a French Canadian population.

## 2. Methods

### 2.1. Study Design

We carried out a two-phase study (i) to translate and culturally adapt the CHAMPS into Canadian French and (ii) to evaluate its clinometric properties (test-retest reliability and convergent construct validity). The results of clinometric testing will be presented in a separate article. In this paper, we are reporting on the methodology and results of the first phase, in which the CHAMPS questionnaire was translated into French and administered to five participants followed by cognitive debriefing. The study was approved by the Research Ethics Board of the McGill University Health Centre (MUHC), and written informed consent was obtained from all participants.

### 2.2. Study Sample

Participants were recruited from the COPD clinic of the Montreal Chest Institute, MUHC. All participants had a primary diagnosis of COPD. Specific inclusion criteria were as follows: (1) age ≥ 40 years, (2) current or previous smoker with smoking history of at least 10 American pack-years, (3) forced expiratory volume in 1 second (FEV_1_) after bronchodilator < 70% of the predicted normal value and a ratio of forced expiratory volume in 1 second to forced vital capacity (FEV_1_/FVC) < 70%, (4) ability to read and understand English or French, and (5) disease stability two weeks prior to enrolment which was defined as no important change in respiratory medications, symptoms, health-related quality of life, and spirometry. Participants were excluded if they had (1) a primary diagnosis of asthma, (2) personal or professional obligations that could cause changes in physical activity practices during the two-week study period (would affect test-rest reliability), or (3) a terminal illness, dementia, or uncontrolled psychiatric illness.

Typically, a sample size of ten individuals is used for questionnaire pretesting stage [21], which includes questionnaire administration and cognitive debriefing. In our study, only five individuals were recruited for pretesting, as there was little variability in participant responses and therefore further recruitment was not necessary.

### 2.3. Translation and Cross-Cultural Adaptation of CHAMPS

**Figure 1:**
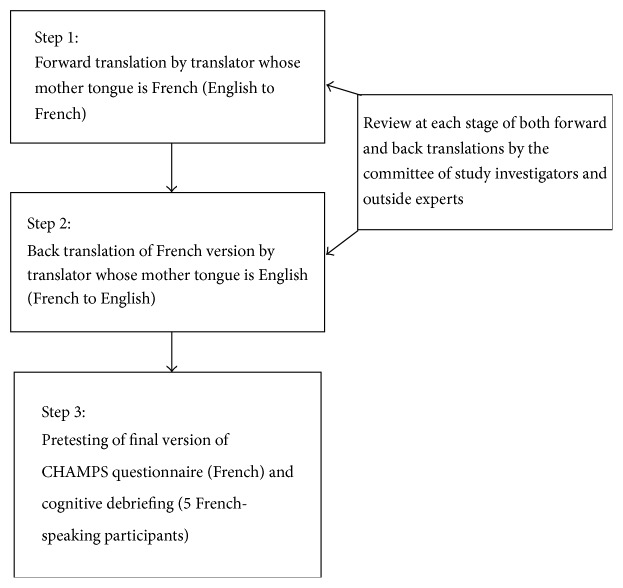
Cross-cultural adaptation process of the CHAMPS questionnaire for the Canadian French COPD population.

Translation and cross-cultural adaptation of the CHAMPS was carried out in 3 steps (Figure 1). Step 1 is* forward translation*. A professional translator who was a native French Canadian speaker and fluent in English translated the original CHAMPS into French. Step 2 is* back translation*. An English Canadian professional translator who was also fluent in French translated the French CHAMPS back into English. The back translator was not aware of the intention of the study but was familiar with COPD.

Several criteria were considered for the selection of translators. For the forward translation into Canadian French, a translator who had not studied in France was chosen to minimize the potential for translation using European French terms or phrases [20]. Familiarity of the translator with the concepts assessed in the questionnaire was also important to ensure optimal equivalence in clinical terminology and meaning between the English and French versions [20]. At each stage of translation (Steps 1 and 2), the forward and back translated questionnaires were reviewed by a committee consisting of the study investigators and outside experts in physical activity and translation methodology. The role of this committee was to verify equivalence between the original English and the translated French versions and to finalize the French version to be used for pretesting [21]. Step 3 is* pretesting and cognitive debriefing.* Five (5) French-speaking participants were recruited from the Montreal Chest Institute COPD Clinic and were asked to complete the French version of the CHAMPS (pretesting). Questionnaire administration was carried out individually with each participant. He/she was given a brief introduction of the study by one of the investigators and was asked to provide sociodemographic information and complete the CHAMPS questionnaire. The study investigator reviewed the instructions of the CHAMPS with each participant and remained available should the participant require any clarification. Following questionnaire completion, each participant completed a one-on-one, cognitive debriefing session where they were asked twelve specific probing questions, developed by the study investigators (Figure 1). The cognitive debriefing used a question and answer format, in order to understand how each participant answered the CHAMPS items. This method contributes to ensuring content validity, which will ultimately minimize measurement error when the French questionnaire is administered in a clinical or research setting. The structure of the cognitive debriefing sessions remained flexible to adapt to participants' responses and allow for additional questions to be asked. One of the investigators (Susanne Mak) conducted the cognitive debriefing sessions, recorded responses, and reviewed responses from all sessions to identify problematic questionnaire items.

## 3. Results

### 3.1. Forward and Back Translation (Steps 1-2)

Following the forward translation of the CHAMPS into French, a review by the expert committee found no problematic items. Once the back translation was conducted, a second review was completed and discrepancies between the original and translated versions were identified in nine items (Table 1). Three items contained errors in translation and were subsequently modified by the translator to more accurately reflect the original items. For example, an original item on the English version states the following: “Do woodworking, needlework, drawing, or other arts or crafts.” In the forward translation, the item was translated in French to the following: “*Fait de la sculpture, du tricot, du dessin ou autre forme d'art ou d'artisanat?*” The activities, “*sculpture*” and “*tricot*,” did not match the activities of woodworking and needlework, respectively. The final translated text for this item was the following: “*Fait de l'ébénisterie, des travaux à l'aiguille, du dessin ou autre forme d'art ou d'artisanat*.”

In the other six items where discrepancies were observed, the translated statements in the French version did not capture the intensity of the physical activity as depicted in the English version. For example, an original item on the English version states the following: “Do light work around the house (such as sweeping or vacuuming).” The translated French version for this item is the following:* “Effectué des tâches ménagères dans la maison (passer le balai, l'aspirateur)*.*”* Following a discussion with the back translator, the committee decided not to modify these items as the activity intensity was implied through the examples depicted in each item.

### 3.2. Pretesting and Cognitive Debriefing (Step 3)

Characteristics of the five participants are provided in Table 2. Participants had a mean age of 64 years and were primarily male ex-smokers. The educational level of participants ranged from 6 to 16 years of schooling. According to the Medical Research Council (MRC) dyspnea scale, two out of five participants described their level of dyspnea as mild-to-moderate (MRC grade: 1–3), while the remaining participants reported severe dyspnea (4-5). The mean FEV_1_ (% predicted) of participants was 36, which, according to the GOLD classification, represents severe COPD (Stage 3) [22]. Spirometry values were missing for one participant.

Of the twelve cognitive debriefing questions administered to the participants (see the questions below), the first four were related to CHAMPS items about playing golf. These items were not applicable as participants either had never played golf or had stopped playing golf. The remaining debriefing questions asked participants to explain specific French terms used in the questionnaire, such as “gros travaux” (heavy work), “travaillé” (worked), and “intensité modérée” (moderate intensity), or to reformulate CHAMPS items in their own words. Since all participant responses indicated good comprehension of French terms and CHAMPS items, further modifications to the French version of the CHAMPS were not necessary.

Cognitive debriefing questions were as follows:

 Q9:
  (i) How easy or difficult was it for you to recall the information to answer this question?
   
*À quel point vous est-il facile ou difficile de vous souvenir de l'information pour répondre à cette question? *(R)^d^
  (ii) How certain are you of your answer?* À quel point êtes-vous certain(e) de votre réponse? *(J)^e^
 Q10:
  (i) How easy or difficult was it for you to recall the information to answer this question?
   
*À quel point vous est-il facile ou difficile de vous souvenir de l'information pour répondre à cette question?* (R)
  (ii) How certain are you of your answer?* À quel point êtes-vous certain(e) de votre réponse?* (J)
 Q19:
  (i) What does the phrase “gros travaux” mean for you?
   
*Qu'est-ce que l'expression “gros travaux” signifie pour vous? *(C)^f^
 Q21:
  (i) What does the phrase “gros travaux” mean for you in the context of this question?
   
*Qu'est-ce que l'expression “gros travaux” signifie pour vous dans le contexte de cette question?* (C)
 Q22:
  (i) What did you think about when answering this question?
   
*À quoi pensiez-vous en répondant à cette question?* (C)
 Q23:
  (i) Rephrase this question in your own words.
   
*Reformulez la question dans vos propres mots.* (C)
  (ii) What does the term “work” mean in the context of this question?
   
*Qu'est-ce que le terme “travailler” signifie dans le contexte de cette question?* (C)
 Q30:
  (i) Rephrase this question in your own words.
   
*Reformulez la question dans vos propres mots.* (C)
 Q32:
  (i) What does “moderate intensity” mean for you?
   
*Que veut dire “intensité modérée” pour vous?* (C)
 Q37:
  (i) What did you think about when answering this question?
   
*À quoi pensiez-vous en répondant à cette question?* (C)
 Q38:
  (i) Rephrase this question in your own words.
   
*Reformulez la question dans vos propres mots.* (C)

^d^R denotes the stage of retrieval in the “question and answer” process, ^e^J denotes the stage of judgment in the “question and answer” process, and ^f^C denotes the stage of comprehension in the “question and answer” process.

## 4. Discussion

The cross-cultural adaptation process allowed for the CHAMPS to be adapted specifically for French Canadians, while preserving the content and structure of the original version. The forward and back translations of the CHAMPS ensured that the meaning of the items was maintained between the English and French versions. The review of the back translation led to slight modifications of three French questionnaire items. Pretesting and cognitive debriefing were carried out in five individuals with a diagnosis of COPD. Responses to cognitive debriefing questions indicated that participants found the French version of the CHAMPS clear and easy to understand. Because the aim of this study was to carry out a structured and stepwise translation process, the following discussion will focus on methodological considerations, strengths, and limitations of this process.

The background of the translators used in the cross-cultural adaptation process was instrumental to maximizing the equivalence between the two versions. Both translators in the forward and back translations were professional translators, whose mother tongue was the language used in the translation [23]. The translator chosen for the forward translation was a native French Canadian speaker so that the terms used in the French CHAMPS would be typical of Canadian French and not European French. For the back translation, the selection of a translator whose mother tongue was the original language of the document was also essential [20]. To maximize integrity and minimize bias, the translator for the back translation had not seen the original English questionnaire [23]. Both translators were also familiar with the concepts examined in the CHAMPS, which enhanced the equivalence in clinical terminology and meaning between the two versions [20].

According to translation methodology, two translators are generally recommended; however, it has been reported that one translator is sufficient [24]. For reasons of cost savings and time, one translator for each stage of translation was employed. The disadvantage of using one translator is the production of a single forward translation that relies completely on the translator's skill and knowledge, possibly leading to low validity and reliability of the measure [25]. In more recent guidelines by the American Association of Orthopedic Surgeons (AAOS), the Mapi Research Institute, and Acquadro et al. [25], the use of at least two translators for each stage of translation has been recommended for measures of health-related quality of life. In our study, the use of an additional translator would have produced a second independent translation and therefore a broader range of French language terms. The limitation of only one translator was likely minimal, however, since the concepts and activities assessed by the CHAMPS are less abstract than those of health-related quality of life. Consequently, the finalized French version of the CHAMPS was produced with few problematic items requiring modification.

Another possible limitation in the translation process was that no formalized instructions were given to the translators other than to translate the CHAMPS questionnaire from English into French or vice versa and to maintain the format of the questionnaire from the original version [25]. Formalized instructions would have ensured consistency of information amongst translators; however, the impact of this limitation was minimal as only one translator was involved at each stage of translation.

The review process by the expert committee at each stage of translation provided another point of verification between the two versions to maximize equivalence. For example, the committee identified translational errors and explained to the translator the meaning of certain questionnaire items, which facilitated correction of those errors. Our committee included experts in the fields of translational methodology, physical activity, and COPD, which contributed to optimizing clinical equivalence and provided valuable insight into the cross-cultural adaptation process.

Although a sample size of ten individuals is often used for the pretesting stage [21], there was little variability in responses of the first five participants and therefore no further recruitment was necessary. Visual inspection of the subject characteristics (Table 2) demonstrated that participants were representative of our target population of moderate-to-severe COPD. There are a higher proportion of males than females in this participant sample, which is consistent with the gender distribution of COPD in Canada [26, 27]. However, the ratio of males (4) to females (1) in our sample is considerably greater than what has been previously reported in the literature (74.3 per 1000 males and 58.2 per 1000 females, or ratio of 1.3 : 1) [26, 27].

In the cognitive debriefing sessions, techniques such as paraphrasing and probing were used to identify potential problematic items [28] and allowed the interviewer to be flexible in her questioning in order to obtain comprehensive responses from the participants. This structure facilitated the interviewer's questioning while remaining organized and consistent amongst participants. The content of some cognitive debriefing questions did not apply to the participants, as several CHAMPS items pertained to the activity of playing golf. According to responses obtained in the cognitive debriefing sessions, the items originally thought by the expert committee to be problematic in the French version were in fact not difficult for participants to understand. It is possible that inclusion of a broader selection of questionnaire items in the cognitive debriefing sessions may have resulted in greater recall and reporting of problematic items.

An informal approach was chosen to document participants' responses from the cognitive debriefing sessions. Although a more rigorous approach is to tape-record these sessions and transcribe all recorded information verbatim, the less formal approach used in this study is commonly used in cross-cultural adaptation of questionnaires [21]. Based on the similarity of participant responses in the cognitive debriefing sessions and the small number of problematic items requiring modification, this limitation likely did not affect the linguistic quality of the finalized French CHAMPS.

The CHAMPS questionnaire is easy to use due to standardized instructions and is feasible due to its administration time of approximately 20 minutes. It also provides information that is highly relevant in both clinical and research settings. At an individual level, clinicians can use the CHAMPS to evaluate the physical activity level of a given patient and to plan appropriate educational and exercise programs aimed at achieving a higher activity level. In a research context, administration of the CHAMPS to large numbers of patients could identify patterns of physical activity levels according to disease stage of COPD. This information could inform the development of disease-specific physical activity guidelines, which could contribute to reducing hospital readmissions [2] and ultimately mortality [1] in this population. A future article will report on the reliability and validity testing carried out on the Canadian French CHAMPS. Future studies should also examine the Canadian French CHAMPS' ability to measure change over time.

## 5. Conclusion

This study illustrated the steps required to translate and culturally adapt the original CHAMPS questionnaire so that it can be used in French for the COPD population in Canada. Through the multiple stages of translation and review in the cross-cultural adaptation process, the meaning of the questionnaire items was maintained and errors in translation were corrected. Pretesting and cognitive debriefing verified that French questionnaire items were well understood and did not require further modification. These steps produced a final French version ready to be evaluated for its clinometric properties.

## Figures and Tables

**Table 1:** Summary of errors noted in the forward translation of CHAMPS based upon committee review.

English version	Translated French version	Comment	Action taken
(4) Attend church or take part in church activities?	Assisté à la messe ou participé aux activités de la paroisse?	Is there a more general term that could be used, such as religious services?	No change

(8) Do woodworking, needlework, drawing, or other arts or crafts?	Fait de la sculpture, du tricot, du dessin ou autre forme d'art ou d'artisanat?	Woodworking and needlework do not match *sculpture *and *tricot*	Changed to: Fait de l'ébénisterie, des travaux à l'aiguille, du dessin ou autre forme d'art ou d'artisanat?

(13) Shoot pool or billiards?	Joué au billard?	Is there another word for billiards or is it the same as pool?	No change

(20) Do light work around the house (such as sweeping or vacuuming)?	Effectué des tâches ménagères dans la maison (passer le balai, l'aspirateur)?	The translated statement does not capture the intensity, “light,” of the tasks	No change

(21) Do heavy gardening (such as spading and raking)?	Effectué de gros travaux de jardinage (bêcher, râcher)?	*Is spading equivalent to digging?*	*No change*

(22) Do light gardening (such as watering plants)?	Jardiné (arroser les plantes)?	Does not capture the intensity of the activity	No change

(25) Walk uphill or hike uphill (count only uphill part)?	Monté des pentes en marchant ou fait de la randonnée pédestre en montagne (calculer seulement le temps de montée)?	Uphill and *montagne *do not match—suggest *monté des pentes en marchant ou en faisant de la randonnée pédestre?*	Changed to: Monté des pentes en marchant ou en faisant de la randonnée pédestre (calculer seulement le temps de montée)?

(30) Do other aerobic machines such as rowing or step machines (do not count treadmill or stationary cycle)?	Fait de l'aérobic en utilisant un appareil à ramer ou les escaliers d'exercice “stairmaster” (ne pas tenir compte du tapis roulant ou du vélo stationnaire)?	Translated question asks about rowing or step machine and **not any other machine**, whereas English version uses them as examples	Changed to: Fait de l'aérobic en utilisant, par exemple, un appareil à ramer ou les escaliers d'exercice “stairmaster” (ne pas tenir compte du tapis roulant ou du vélo stationnaire)?

(38) Do light strength training (such as hand-held weights) of 5 lbs or less or elastic bands?	Fait des exercices de force musculaire à faible intensité (levee de poids de moins de 5 livres (2.6 kg) ou bande élastique)?	*Faible intensité*—does it truly capture the term “light” or could the term “légère” be used?	No change

**Table 2:** Characteristics of participants in pretesting and cognitive debriefing (*n* = 5).

Characteristic	Participants	
	Mean (SD)	*N* (%)
Age	64 (6)	

Sex		
Female		1 (20)
Male		4 (80)

Smoking status		
Ex-smoker		4 (80)
Current smoker		1 (20)

Dyspnea^a^		
Mild-to-moderate		3 (60)
Severe		2 (40)

FEV_1_ (L)^b^	1.0 (0.78)	

FEV_1_/FVC (%)^c^	37 (11)	

FEV_1_ (% predicted)	36 (24)	

Number of comorbid conditions		
0		2 (40)
1		2 (40)
2-3		1 (20)

^a^Dyspnea measured by the modified Medical Research Council dyspnea scale: mild-to-moderate (1–3) and severe dyspnea (4-5).

^b^ *n* = 4 for spirometry.

^c^ *n* = 4 for spirometry.
